# Evaluation of a Pre-Filled Table and a Flowchart-Based Algorithm as Cognitive Aids to Reduce Deviations in Dose Calculation for Intraoperative Red Blood Cell Transfusions in Children—An International Web-Based Simulation

**DOI:** 10.3390/children10050815

**Published:** 2023-04-29

**Authors:** Florian Piekarski, Stephanie Noone, Thomas Engelhardt, Martin Hellmich, Eva Wittenmeier, Vinicius Quintao, Philip Arnold, Susan M. Goobie, Kai Zacharowski, Jost Kaufmann

**Affiliations:** 1 Department of Anaesthesiology, Intensive Care Medicine and Pain Therapy, University Hospital Frankfurt, Goethe University, 60590 Frankfurt, Germany; 2 Department for Anesthesia, Montreal Children’s Hospital, Montreal, QC 1001, Canada; 3 Institute for Medical Statistics, Informatics, and Epidemiology (IMSIE), University Hospital Cologne, University of Cologne, 50923 Cologne, Germany; 4 Department of Anesthesiology, University Medical Center of the Johannes Gutenberg-University, 55131 Mainz, Germany; 5 Discipline of Anesthesiology, Hospital das Clínicas HCFMUSP, Faculdade de Medicina, Universidade de São Paulo, São Paulo 05403-010, Brazil; 6 The Jackson Rees Department of Anaesthesia, Alder Hey Children’s Hospital, Liverpool L12 2AP, UK; 7 Department of Anesthesiology, Critical Care and Pain Medicine, Harvard Medical School, Boston Children’s Hospital, Boston, MA 02115, USA; 8 Department for Paediatric Anaesthesia, Children’s Hospital Cologne, 50735 Cologne, Germany; 9 Faculty for Health, University of Witten/Herdecke, 58455 Witten, Germany

**Keywords:** children, transfusion, cognitive aids, red blood cell, simulation, blood loss

## Abstract

Background: Transfusion of red blood cell concentrate can be life-saving, but requires accurate dose calculations in children. Aims: We tested the hypothesis that cognitive aids would improve identification of the correct recommended volumes and products, according to the German National Transfusion guidelines, in pediatric transfusion scenarios. Methods: Four online questionnaire-based scenarios, two with hemodynamically stable and two with hemodynamically unstable children, were sent to German and international pediatric anesthetists for completion. In the two stable scenarios, participants were given pre-filled tables that contained all required information. For the two emergency scenarios, existing algorithms were used and required calculation by the user. The results were classified into three categories of deviations from the recommended values (DRV): DRV120 (<80% or >120%), as the acceptable variation; DRV 300 (<33% or >300%), the deviation of concern for potential harm; and DRV 1000 (<10% or >1000%), the excessive deviation with a high probability of harm. Results: A total of 1.458 pediatric anesthetists accessed this simulation questionnaire, and 402 completed questionnaires were available for analysis. A pre-filled tabular aid, avoiding calculations, led to a reduction in deviation rates in the category of DRV120 by 60% for each and of DRV300 by 17% and 20%, respectively. The use of algorithms as aids for unstable emergencies led to a reduction in the deviation rate only for DRV120 (20% and 15% respectively). In contrast, the deviation rates for DRV300 and DRV1000 rose by 37% and 16%, respectively. Participants used higher transfusion thresholds for the emergency case of a 2-year-old compromised child than for the stable case with a patient of the same age (on average, 8.6 g/dL, 95% CI 8.5–8.8 versus 7.1 g/dL, 95% CI 7.0–7.2, *p* < 0.001) if not supported by our aids. Participants also used a higher transfusion threshold for unstable children aged 3 months than for stable children of the same age (on average, 8.9 g/dL, 95% CI 8.7–9.0 versus 7.9 g/dL, 95% CI 7.7–8.0, *p* < 0.001). Conclusions: The use of cognitive aids with precalculated transfusion volumes for determining transfusion doses in children may lead to improved adherence to published recommendations, and could potentially reduce dosing deviations outside those recommended by the German national transfusion guidelines.

## 1. Introduction

Hypovolemia from massive blood loss is a major cause of perioperative cardiac arrest in pediatric surgery and anesthesia [1]. Prompt and sufficient allogeneic blood product transfusion is often necessary to restore the oxygen transport capacity, maintain tissue oxygenation, maintain hemodynamics, and avoid cardiovascular failure. However, the efficacy and safety of blood transfusion remain concerns [2,3].

Several expert consensuses, evidence-based pediatric blood product transfusion guidelines, and patient blood management algorithms have been published [4,5,6,7]. In children, age-related standard values for hemoglobin (Hb), estimated blood volumes (EBV), transfusion thresholds, and body weight must be considered. Decision making regarding accurate dosage calculations is complex, especially during an unexpected pediatric major hemorrhage. Individual calculation of the amount of red blood cells to be transfused can lead to deviations, which is reflected by the disproportionately high rate of under- and over-transfusion in children [8]. Several algorithms have been proposed to reduce the incidence of erroneous transfusions. Thus, for example, the introduction of an objective transfusion algorithm in pediatric cardiac surgery led to a significant reduction in perioperative blood product consumption and mortality [9]. However, additional cognitive aids may help to further reduce deviations outside the recommended guidelines for pediatric transfusion, especially in emergencies [10].

The German transfusion guidelines, valid at the time of this study, served as the basis. The guideline recommended a hematocrit limit of 20% (6.8 g/dL) for hemodynamically stable and 30% (10.2 g/dL) for unstable pediatric patients older than 4 months. For children aged 28 days to 4 months, a hematocrit cutoff value of 25% (8.5 g/dL) was suggested for stable scenarios [7]. We hypothesized that a cognitive aid could help in determining accurate transfusion thresholds, as well as the optimal volume of red blood cell (RBC) transfusions in children undergoing surgery. To test this hypothesis, we used a simulation questionnaire administered to pediatric anesthetists with and without cognitive aids.

## 2. Materials and Methods

**Figure 1:**
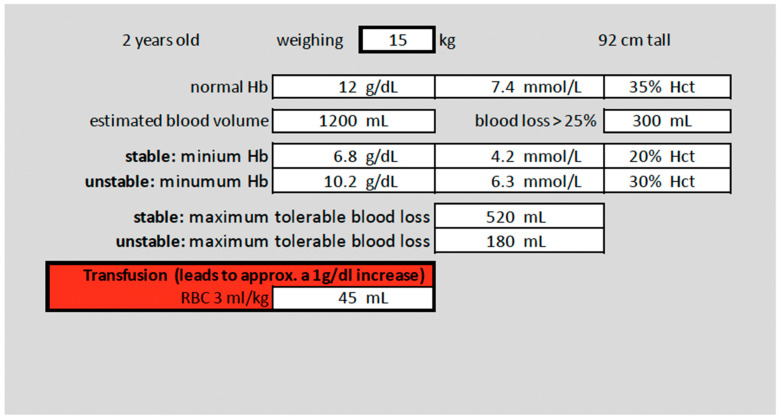
Pre-filled table for stable scenarios.

An anonymized web-based simulation questionnaire with four different clinical scenarios of pediatric anesthesia was developed. All participants were supposed to work on all scenarios, with and without aids. The questionnaire was available to participants in German and English. Two of those scenarios described a child under stable hemodynamic conditions, and two other scenarios described a hemodynamically compromised child (Table 1). The first part of the questionnaire queried the type of institution of the participant’s own affiliation, as well as their level of training and experience in pediatric anesthesia. The second part of the questionnaire asked the participant to determine transfusion thresholds (TTs) and transfusion volumes (TVs) required to achieve an increase of approximately 1 g/dL and the maximum tolerated blood loss (MTBL) without aids. The third part contained the same patient cases as in part 2. Prefilled tables for stable cases and algorithms for unstable patients were provided as aids. Cognitive, pre-filled tables that did not require any calculations were provided for the two stable scenarios (Figure 1).

Figure 1 shows the age-adapted pre-filled table for the scenarios of a stable 2-year-old child.

**Figure 2:**
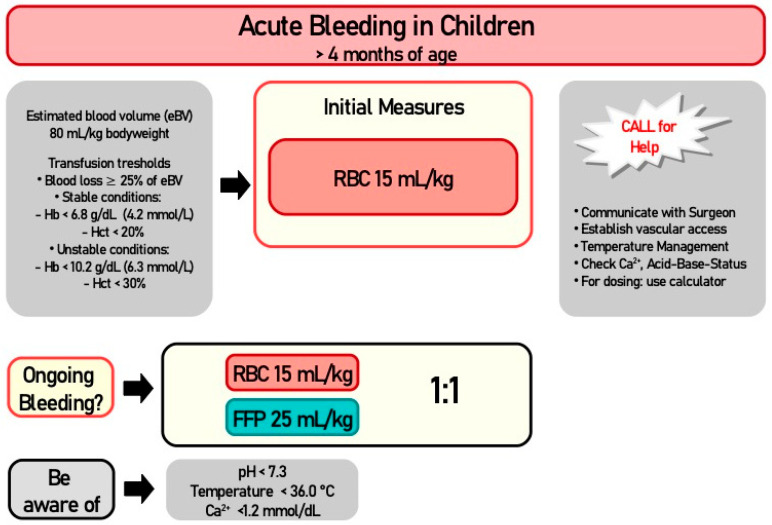
Flow chart algorithm for unstable clinical scenarios.

For the two emergency scenarios, as these may occur unexpectedly, the TT and TV for initial stabilization were requested using an algorithm, and had to be calculated by the participants themselves (Figure 2). The questionnaire was tested in a pilot project and optimized, although only in technical, and not in content, aspects, after feedback from 10 participants. 

Figure 2 shows the cognitive aid for the unstable situation adapted to children over 4 months of age. It is a flow chart algorithm for clinical scenarios with acute bleeding.

### 2.1. Normal Value Definitions and Calculations

Definitions of normal values were derived from the German transfusion guidelines [7]. The maximum tolerated blood loss was calculated as MTBL = EBV × (Hbstart − Hbthreshold)/Hbstart. The values set as correct for this study are shown in the case descriptions in Table 1. The classification methodology of deviations from the recommended values (DRV) is well established in drug safety research [11,12]. The DRV classification was adopted for this study:

DRV120 (<80% or >120%): Interpreted as a deviation that is considered tolerable or unlikely to cause health concerns, since the deviation is within the range of other transfusion threshold recommendations or is the result of other formulas for calculating transfusion volumes.

DRV300 (<33% or >300%): Deviation of concern; harm to health due to under- or overdosage may occur.

DRV1000 (<10% or >1000%): Excessive deviation means a high probability of harm to health (overdose) or of not achieving the intended effect (underdose).

### 2.2. Recruitment of Participants, Evaluation of Data, and Statistical Analysis

A link to the web-based simulation questionnaire was provided via e-mail lists to all registered members of the anesthesiology societies with pediatric practice in Germany (DGAI) and Brazil (SBA), as well as the societies for pediatric anesthesia of Europe (ESPA), Canada (CPAS) and the Association of Paediatric Anaesthetists of Great Britain and Ireland (APAGBI). The questionnaire was accessible online from 19 October 2020 to 13 July 2021. One reminder was sent out. The data were collected via the survey portal and exported into a spreadsheet. Only completed questionnaires were considered for analysis. When a response offered a range, the mean of the range was used. If a response indicated a unit of packed RBC instead of the volume, the unit was assumed to contain 300 mL RBC. For descriptive analysis, the open-source software Python 3.1 (The Python Software Foundation) was used. Further calculations were made with spreadsheet software Microsoft EXCEL 365 (Microsoft Corporation, Redmond, Washington, DC, USA) and IBM^®^ SPSS^®^ Statistics Version 26, (IBM^®^, Armonk, New York, NY, USA). Results were presented using absolute and relative frequency. A two-sided Student’s *t* test was used to compare average transfusion thresholds in children of the same age according to whether the situation was stable or unstable. The z-test was used to compare the proportional deviation rates without or with tabular aids [13]. A *p*-value < 0.05 was considered significant. Where appropriate, results were reported with a 95% confidence interval.

### 2.3. Endpoints

The primary endpoint was the impact of cognitive aids on the accuracy of the transfusion dosage. The secondary endpoint was the accuracy transfusion threshold determination, as well as the MTBL, in children undergoing scheduled surgery under stable cardiovascular conditions.

## 3. Results

**Figure 3:**
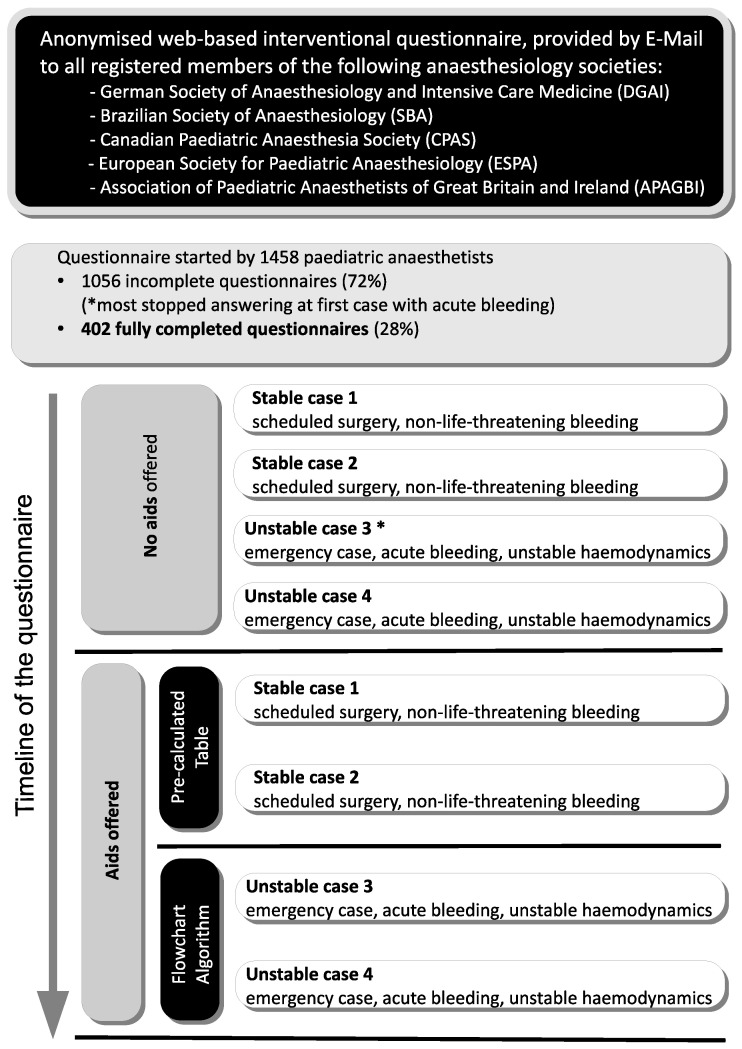
Study flow chart.

A total of 1458 pediatric anesthetists accessed this questionnaire, and 402 (27.6%) completed questionnaires were available for analysis (Figure 3). Participants came from Germany (73.9%), Brazil (9.0%), the United Kingdom (4.7%), Switzerland (2.0%), and 1.2% each from Canada, Austria, and Sweden.

### 3.1. Pre-Filled Table in Stable Scenarios

The pre-filled table aid led to a reduction in deviations from the transfusion dosage in both hemodynamically stable scenarios (DRV120 by 60% in both scenarios and for DRV300 by 17% and 20%, respectively (Table 2)).

**Figure 4:**
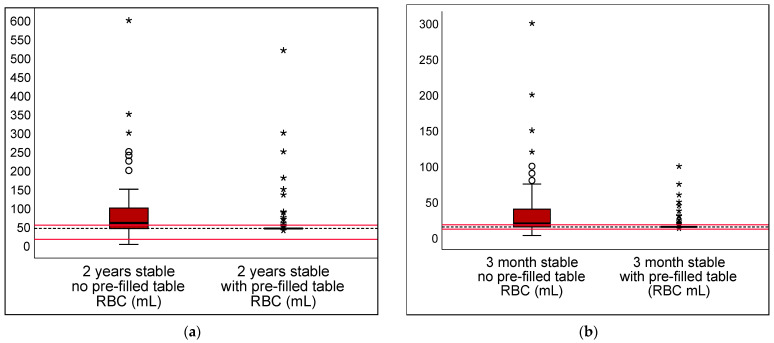
Transfusion volume in stable scenarios, unaided or supported by a pre-filled table. The transfusion volumes in the stable scenarios are shown for 2 year (**a**) and 3 month (**b**) old children. A pre-filled table was provided as an aid in the scenarios involving stable patients. The dotted line shows the calculated reference value. The range of DRV 120 is marked with the red lines. Mild outliers are represented by dots °; extreme outliers by asterisks *.

**Figure 5:**
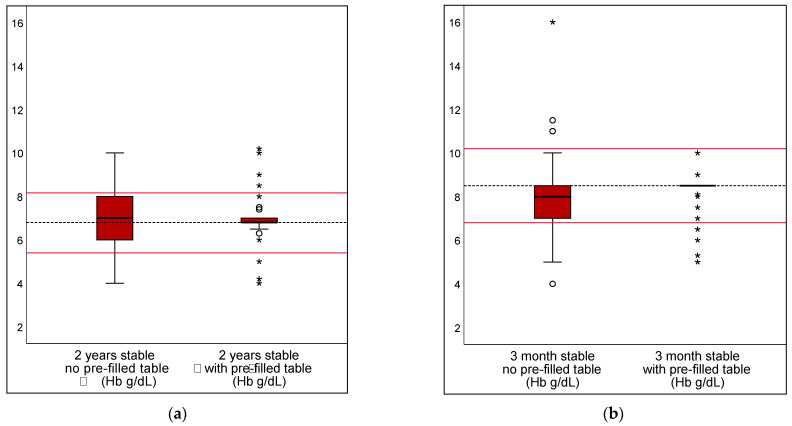
Transfusion threshold in stable scenarios, both unaided and supported by a pre-filled table. Transfusion thresholds in the stable scenarios are shown for 2 year (**a**) and 3 month (**b**) old children. A pre-filled table was provided as an aid in scenarios involving stable patients. The dotted line shows the calculated reference value. The range of DRV 120 is marked with red lines. Mild outliers are represented by dots °; extreme outliers by asterisks *.

The pre-filled table aids led to a reduction in transfusion threshold deviations from the guideline with DRV120 by 7% and 15% in children under stable conditions. No significance was found in the evaluation of DRV300 or DRV1000 (Table 2). Figure 4 and Figure 5 show the transfusion volumes and thresholds in stable scenarios, unaided or supported by a pre-filled table, using boxplots.

For the stable cases, MTBL was queried at the baseline. Here, the cognitive table aids resulted in a reduction in deviation from the guideline values of 45% for DRV120 and 10% for DRV300 in the 3-month-old hemodynamically stable child, and of 49% for DRV120 and 7% for DRV300 in the 2-year-old stable child.

### 3.2. Algorithm in Unstable Scenarios

**Figure 6:**
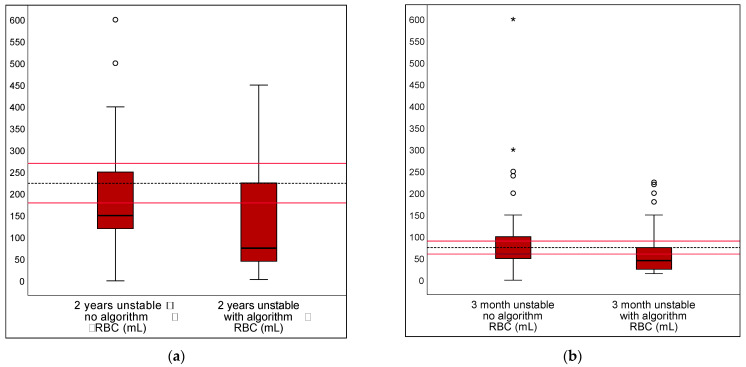
RBC bolus for initial stabilization in unstable scenarios, unaided or supported by an algorithm. RBC bolus for initial stabilization in cases of ongoing hemorrhage are shown for (**a**) 2 year old and (**b**) 3 month old children. As an aid in scenarios involving unstable patients, an algorithm requiring a calculation step by the user was made available. The dotted line shows the calculated reference value. The range of DRV 120 is marked with red lines. Mild outliers are represented by dots °; extreme outliers by asterisks *.

**Figure 7:**
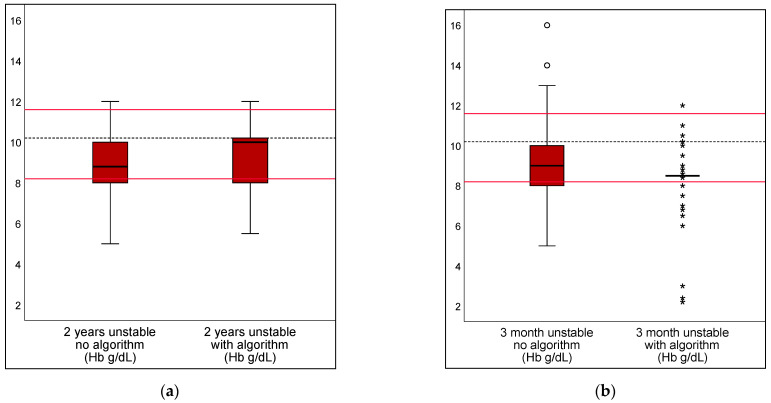
Transfusion thresholds in unstable scenarios, unaided or supported by an algorithm. Transfusion thresholds in the unstable scenarios are shown (**a**) for 2 year and (**b**) 3 month old children. As an aid for scenarios involving unstable patients, an algorithm requiring a calculation step by the user was made available. The dotted line shows the calculated reference value. The range of DRV 120 is marked with red lines. Mild outliers are represented by dots °; extreme outliers by asterisks *.

The algorithm provided for the hemodynamically unstable children (requiring calculations) resulted in a reduction in deviation of the transfusion dosage only for DRV120 (20% and 15% for each scenario). Deviations for DRV300 rose by 37% and 16% for the two scenarios. The algorithm led to a reduction in transfusion threshold deviations from the guidelines for DRV120 by 23% and 22% under unstable conditions. No significance was found in the evaluation of DRV300 or DRV1000 (Table 2). Figure 6 and Figure 7 show the transfusion volumes and thresholds in unstable scenarios, unaided or supported by the algorithm, using boxplots.

### 3.3. Experience

Thirty-five residents, 21 fellows, 201 consultants, 134 senior consultants, and 11 heads of departments completed the survey. A large proportion (77%, n = 311) of participants had over 5 years’ experience in pediatric anesthesia in children under 8 years. Most participants were affiliated with university hospitals (34%, n = 137), whereas 10% (n = 38) worked in specialized pediatric hospitals. We did not observe any significant impacts of the experience levels or origins of the participants on the error rates or the influence by the cognitive aids.

### 3.4. Transfusion Thresholds

Participants used higher transfusion thresholds for the emergency case of a 2-year-old compromised child than for the hemodynamically stable scenario involving a child of the same age (mean 8.6 g/dL, 95% CI 8.5–8.8 versus 7.1 g/dL, 95% CI 7.0–7.2, *p* < 0.001) if no cognitive aids were used. Participants also used a higher transfusion threshold for hemodynamically unstable children aged 3 months than for stable children of the same age (mean 8.9 g/dL, 95% CI 8.7–9.0 versus 7.9 g/dL, 95% CI 7.7–8.0, *p* < 0.001).

Participants chose significantly higher transfusion thresholds without pre-filled table aids for the scenario of a 3-month-old hemodynamically stable child compared with that of a 2-year-old stable child (7.9 g/dL (95% CI 7.7–8.0) versus 7.1 g/dL (95% CI 7.0–7.2, *p* < 0.001)). This was also seen in the unstable situation, with 8.9 g/dL (95% CI 8.7–9.0 g/dL) versus 8.6 g/dL (95 CI 8.5–8.8 g/dL, *p* < 0.001).

## 4. Discussion

The current study reports the benefits of a pre-filled table aid and an algorithm aid in reducing deviations from the acceptable guidelines in pediatric transfusion scenarios, and underlines the effectiveness of simple tabular aids. It emphasizes that calculation steps must be avoided in all cognitive aids whenever possible. This recommendation is consistent with advice from the pediatric European Resuscitation Council guidelines on medication safety: “Use whenever possible, decision aids providing pre-calculated dose advice for emergency drugs and materials” [14]. This is particularly evident in our study in the comparison of the consistent error reduction achieved through the use of the prefilled table in contrast to the algorithm that required calculation steps.

The transfusion of blood products is a highly challenging task, both medically and legally. Transfusion of the wrong blood products, as well as over- and undertransfusion, can cause severe harm and even death to pediatric patients. The specific indications and timing for the administration of blood products must be unambiguous. These difficult decisions are commonly made during unpredictable scenarios with severe acute hemorrhage during surgery, and even the most qualified and experienced physicians admit that they still have uncertainties regarding the calculation. In addition, pediatric transfusion requires knowledge of age-related values, determined by individual calculations.

The German transfusion guidelines served a basis for this study, and were updated during the current project. The previous guidelines recommended, in children older than 4 months, a transfusion hematocrit threshold of 20% (6.8 g/dL) for hemodynamically stable and 30% (10.2 g/dL) for unstable patients [7]. For children aged 28 days to 4 months, a hematocrit threshold of 25% (8.5 g/dL) was proposed for stable scenarios [7]. In this study, we observed that for both the stable and unstable scenarios, higher transfusion thresholds were used, consistently with the old guidelines. The updated German transfusion guidelines no longer differentiate between children beyond the neonatal period, and recommends that a decrease in the hemoglobin concentration to 6–7 g/dL be used, as this can be tolerated in hemodynamically stable children with adequate volume resuscitation. In unstable, bleeding children, a hemoglobin concentration of 10 g/dL is still recommended in the German guidelines [15]. This distinction is important in the interpretation of our data, since nearly three out of four respondents were from Germany. The recommended transfusion threshold recommendations vary widely across the world. The European Society of Anaesthesiology (ESAIC) advises against transfusion if the child is hemodynamically stable and has an Hb concentration of at least 7 g/dL. For unstable children, the guideline refers to the recommendations of the Transfusion and Anemia Expertise Initiative [16]. The Pediatric Critical Care Transfusion and Anemia Expertise Initiative recommends that in critically ill children with non-life-threatening bleeding, a red blood cell transfusion should be given if the hemoglobin concentration is <5 g/dL, and should only be considered if the hemoglobin concentration is between 5–7 g/dL. In critically ill children with hemorrhagic shock, a transfusion ratio of red cells, plasma, and platelets of 2:1:1 to 1:1:1 is recommended until the bleeding is no longer life-threatening [5]. The British Guidelines from 2016 recommend a transfusion threshold of 7 g/dL for stable children undergoing non-cardiac surgery and a target Hb of 8 g/dL in a massive hemorrhage situation [17]. The Australian Patient Blood Management guideline, currently under revision, also maintains a transfusion threshold of <7 g/dL for hemodynamically stable children. In addition, it explicitly points out the importance of calculating the transfusion volume [18]. Since the assessment of the transfusion threshold provided in the questionnaire was judged to be correct within a 20% deviation, the described differences due to variations in the guidelines alone did not trigger an error. Our study showed that, on average, participants followed, in general, the recommendations for the hemodynamically stable scenarios, and were restrictive in critical scenarios. Importantly, there were relevant outliers in both under- and overtransfusion, which may be associated with a negative outcome in a real patient.

The current study shows that a pre-filled table aid can potentially reduce deviations when calculations are eliminated from the process and the amounts of volumes to be transfused are precalculated. This is especially beneficial during emergencies and acute hemorrhage scenarios. Apart from an electronic aid, as used in this study, other potential solutions are conceivable. For instance, a spreadsheet could be prepared in which weight, age, and starting hemoglobin have been entered preoperatively and all relevant data are available in an emergency. A visually focused display, in the form of an application with key selection categories, such as massive transfusion protocol, may also lead to a reduction in deviation. Other options include prepared algorithms with standardized weights. Computerized physician order entry (CPOE) systems have a proven positive impact on drug safety [19]. Such systems adopted for transfusion needs are desirable, and should particularly consider age- and condition-related recommendations.

Standardization in the real world must also consider the influence of the individual facility and the experience of the anesthesiologist and surgeon. In particular, transfusion thresholds may also change in real life during the course of resuscitation according to the bleeding rate, duration of the procedure, volume substitution, volume responsiveness, and other factors. These real-life factors play an important role in the success or failure of transferring the results of cognitive tools from simulation to real life. However, a successful transfer to real life might be initiated due to our findings, and has already been achieved elsewhere. As an example, regarding the real-life implementation of cognitive aids, the Pediatric Emergency Ruler (PaedER; www.paeder.com accessed on 17 February 2023) has been proven to reduce dosing errors in pediatric emergencies. The use of the PaedER reduced the rate of severe dosing errors (with a deviation from the recommended doses larger than 300%) by 80% across all studied drugs, and by 100% for epinephrine [20].

This current study has several limitations. Like other studies looking at pharmacological dosing errors, there are no clear and defined margins of error for transfusion dosing. Most available drug studies assume that a deviation of 20% is erroneous. However, based on clinical considerations, such a limit is relevant neither for drug dosing nor for transfusion dosing. For both questions, however, deviations by a factor of three appear to be life-threatening for pediatric patients, and tenfold errors are very likely to be associated with serious adverse effects.

Another limitation is that outside Germany, these cognitive aids do not follow the recommendations of the expert consensus guidelines mentioned above. Participants from outside Germany may follow the more restrictive recommendations of their countries and be misclassified as deviating from the guidelines. However, it must be stated that deviations above DRV120 do not correspond to the recommendations of other guidelines.

It is possible that deviation rates were underestimated due to selection bias caused by the high proportion of participants with more than 5 years of experience in pediatric anesthesia, and by only enthusiastic and concerned clinicians participating and completing the questionnaire. It is possible that the deviation reduction effects may have been more pronounced in a less selective group. In addition, the questionnaire did not provide the real-life pressure experienced while providing care to an actual unstable child. It should be pointed out herein that cognitive aids should, ideally, be studied in either an actual clinical environment or, failing that, in an in situ simulation in the clinical workplace. Another major limitation of the study with which participants were not familiar was the cognitive aids, whereas in real-life scenarios, the user should be aware of these. The lack of training on the aid was especially relevant for the flow chart algorithm, which required calculation by the participant. This was especially evident in the deviation of DRV300 in unstable scenarios, where unexpected increases of 37% and 16% was observed. Prior training could also likely be better implemented in a clinical trial. Furthermore, just under one-third of the participants fully completed the questionnaire. It is possible that participants were unsure as to whether they would be able to give a correct answer and aborted the task. Those who continued might have been more familiar with the scenario and, therefore, better positioned to estimate and calculate the transfusion volume needed. A total of 18 participants reported the transfusion volume in units other than ml; in the present study, an RBC unit was assumed to contain 300 mL. However, the content of an RBC unit can range from 200 to 350 mL.

## 5. Conclusions

The use of cognitive aids with precalculated transfusion volumes for determining red blood cell transfusion doses in children may lead to improved dosing and the avoidance of dosing deviations outside acceptable guidelines in a simulated online questionnaire. Such cognitive aids should be provided, especially in pediatric patients with a high risk of hemorrhage. This findings needs to be confirmed in an actual clinical environment.

## Figures and Tables

**Table 1:** Scenarios used with stable and unstable conditions.

Short Name	Case	Preexisting Conditions	Calculations: Values Used as Correct
2 years, stable	2-year-old female, 15 kg body weight, scheduled surgery: partial spleen resection	Bronchial asthma, ASA Classification 2, stable hemodynamics, preoperative Hb-level 12 g/dL	- MTBL 520 mL - Transfusion threshold Hb 6.8 g/dL - RBC (mL) required for Hb increase of 1 g/dL (3 mL/kg): 45 mL
3 months, stable	3-month-old male, 5 kg body weight, scheduled surgery: pull-through procedure (Hirschsprung’s disease)	No pre-existing cardiovascular conditions, born at term, stable hemodynamics, preoperative Hb-level 13 g/dL	- MTBL 138 mL - Transfusion threshold Hb 8.5 g/dL - RBC (mL) required for Hb increase of 1 g/dL (3 mL/kg): 15 mL
2 years, unstable	2-year-old male, 15 kg body weight, emergency neurosurgical treatment of traumatic epidural hemorrhage, relevant bleeding situation and continuous blood loss after craniotomy	No pre-existing conditions known; currently impaired cardiovascular conditions due to acute bleeding	- Transfusion threshold Hb 10.2 g/dL - RBC (ml) required for initial stabilization (15 mL/kg): 225 mL
3 months, unstable	3-month-old male, 5 kg body weight, scheduled surgery: hemi-hepatectomy (congenital hepatoblastoma), relevant blood loss due to complications	No pre-existing cardiovascular conditions, born at term; currently impaired cardiovascular conditions due to acute bleeding	- Transfusion threshold Hb 10.2 g/dL - RBC (mL) required for initial stabilization (15 mL/kg): 75 mL

MTBL = maximal tolerable blood loss; RBC = red blood cell concentrate.

**Table 2:** Deviations from the recommended values.

Deviation Grade	Unaided	Aided	Diff	95% CI	*p*
*2 years, 15 kg, preop Hb 12 g/dL; splenectomy, stable conditions*					
Hb transfusion threshold; correct value set as 6.8 g/dL					
DRV120	13.4%	6.2%	7.2%	2.9–11.5%	0.0011
DRV300	0.2%	0.0%	0.2%	−0.2–0.7%	0.3173
DRV1000	1.7%	0.0%	0.2%	−0.2–0.7%	0.3173
RBC transfusion volume; correct value set as 45 mL					
DRV120	66.7%	6.5%	60.2%	51.8–68.6%	<0.001
DRV300	18.9%	1.7%	17.2%	12.7–21.6%	<0.001
DRV1000	1.0%	0.5%	0.5%	−0,7–1,7%	0.4142
MABL; correct value set as 520 mL					
DRV120	72.4%	27.9%	44.5%	34.7–54.3%	<0.001
DRV300	11.4%	1.7%	9.7%	6.2–1.3%	<0.001
DRV1000	1.7%	0.0%	1.7%	0.5–3.0%	0.0082
Deviation grade	unaided	aided	Diff	95% CI	*p*
*3 months, 5 kg, preop Hb 13 g/dL; rectal pull-through, stable conditions*					
Hb transfusion threshold; correct value set as 8.5 g/dL					
DRV120	19.9%	4.7%	15.2%	10.3–20.0%	<0.001
DRV300	0.2%	0.7%	−0.5%	−1.5–0.5%	0.3173
DRV1000	0.0%	0.2%	−0.2%	−0.7–0.2%	0.3173
RBC transfusion volume; correct value set as 15 mL					
DRV120	69.7%	9.5%	60.2%	51.5–68.9%	<0.001
DRV300	21.4%	1.2%	20.1%	15.5–24.8%	<0.001
DRV1000	1.2%	0	1.2%	0.2–2.3%	0.0253
MABL; correct value set as 138 mL					
DRV120	73.9%	24.6%	49.3%	39.6–59.0%	<0.001
DRV300	7.2%	0.2%	7.0%	4.3–9.6%	<0.001
DRV1000	0.5%	0.2%	0.2%	−0.6–1.1%	0.5637
Deviation grade	unaided	aided	Diff	95% CI	*p*
*2 years, 15 kg; bleeding after major surgery, unstable hemodynamic condition*					
Hb transfusion threshold; correct value set as 10.2 g/dL					
DRV120	49.8%	27.1%	22.6%	14.1–31.2%	<0.001
DRV300	0.5%	0.5%	0.0%	−1.0–1.0%	10000
DRV1000	0.0%	0.5%	−0.5%	−1.2–0.2%	0.1573
RBC transfusion volume; correct value set as 225 mL					
DRV120	82.6%	61.7%	20.9%	9.2–32.6%	<0.001
DRV300	10.4%	47.3%	−36.8%	−44.2–−29.4%	<0.001
DRV1000	2.0%	3.0%	−1.0%	−3.2–1.2%	0.3711
Deviation grade	unaided	aided	Diff	95% CI	*p*
*3 months, 5 kg; bleeding after major surgery, unstable hemodynamic condition*					
Hb transfusion threshold; correct value set as 10.2 g/dL					
DRV120	43.8%	21.9%	21.9%	14.0–26.9%	<0.001
DRV300	0.5%	2.0%	−1.5%	−3.0–0.0%	0.0578
DRV1000	0.5%	1.0%	−0.5%	−1.7–0.7%	0.4142
RBC transfusion volume; correct value set as 75 mL					
DRV120	82.1%	67.2%	14.9%	3.0–26.9%	0.0143
DRV300	8.7%	24.6%	−15.9%	−21.6%–−10.3%	<0.001
DRV1000	0.7%	0.0%	0.7%	−0.1–1.6%	0.0833

Diff = difference; DRV = deviation from correct value; MABL = maximum tolerated blood loss.

## Data Availability

The data that support the findings of this study are available from the corresponding author upon reasonable request.
